# Chemical genomics reveals inhibition of breast cancer lung metastasis by Ponatinib via c-Jun

**DOI:** 10.1007/s13238-018-0533-8

**Published:** 2018-04-17

**Authors:** Wei Shao, Shasha Li, Lu Li, Kequan Lin, Xinhong Liu, Haiyan Wang, Huili Wang, Dong Wang

**Affiliations:** 1 Department of Basic Medical Sciences, School of Medicine, Tsinghua University, Beijing, 100084 China; 2 Collaborative Innovation Center for Biotherapy, West China Hospital, Sichuan University, Sichuan, 610041 China; 3 Center for Synthetic and Systems Biology, Tsinghua University, Beijing, 100084 China

**Keywords:** anti-metastatic drug discovery, gene expression signature, high-throughput sequencing-based high-throughput screening, Ponatinib, breast cancer lung metastasis, c-Jun

## Abstract

Metastasis is the leading cause of human cancer deaths. Unfortunately, no approved drugs are available for anti-metastatic treatment. In our study, high-throughput sequencing-based high-throughput screening (HTS^2^) and a breast cancer lung metastasis (BCLM)-associated gene signature were combined to discover anti-metastatic drugs. After screening of thousands of compounds, we identified Ponatinib as a BCLM inhibitor. Ponatinib significantly inhibited the migration and mammosphere formation of breast cancer cells *in vitro* and blocked BCLM in multiple mouse models. Mechanistically, Ponatinib represses the expression of BCLM-associated genes mainly through the ERK/c-Jun signaling pathway by inhibiting the transcription of *JUN* and accelerating the degradation of c-Jun protein. Notably, *JUN* expression levels were positively correlated with BCLM-associated gene expression and lung metastases in breast cancer patients. Collectively, we established a novel approach for the discovery of anti-metastatic drugs, identified Ponatinib as a new drug to inhibit BCLM and revealed c-Jun as a crucial factor and potential drug target for BCLM. Our study may facilitate the therapeutic treatment of BCLM as well as other metastases.

**Electronic supplementary material:**

The online version of this article (10.1007/s13238-018-0533-8) contains supplementary material, which is available to authorized users.

## Introduction

The absence of effective and high-throughput metastatic cell models *in vitro* has obstructed anti-metastatic drug discovery. Metastasis represents a multistep cascade of events, including local invasion, intravasation, survival in the circulation, extravasation and colonization (Nguyen et al., 2009). Few *in vitro* cell assays can be used to represent a particular step of metastasis. For example, Transwell migration or wound healing assays can be used to study cell migration or invasion; and soft-agar colony-formation assays are a well-established method for characterizing cell colonization capability. However, these assays cannot completely reflect the cancer metastatic process and are not suitable for anti-metastatic drug discovery.

Breast cancer is the most common invasive cancer in women (Siegel et al., 2017), and the lungs are one of the most common metastatic organs (Lee, 1983; Quiet et al., 1995). Despite enormous efforts in academia and industry to develop specifically anti-metastasis medications for breast cancer, no drugs are available on the market. Therefore, there is an urgent needed for effective medical treatments for breast cancer metastasis.

The gene expression signatures of cells or tissues can be used as “fingerprints” to define cancer subtyping (Perou et al., 2000), predict cancer metastasis (Kang et al., 2003; Minn et al., 2005; Bos et al., 2009) and determine the clinical outcome of patients (van’t Veer et al., 2002). Gene expression profiling of cellular perturbations has been used to effectively predict drug sensitivity (Chambers et al., 1999; Ayers et al., 2004) and compound mechanism-of-action (MoA) (Iorio et al., 2010), as well as for anti-cancer drug discovery (Stegmaier et al., 2004; Lamb et al., 2006; Li et al., 2012b; Lee et al., 2016). Compared with common technologies for detecting gene expression, such as microarray (Lamb et al., 2006) and Luminex beads (Subramanian et al., 2017), high-throughput sequencing-based high-throughput screening (HTS^2^), which takes advantage of powerful next-generation sequencing technologies and greatly enhances the parallel processing of samples and genes (Li et al., 2012b), has a huge advantage in terms of throughput, required labor and costs. Thus, the combination of metastasis-associated gene signatures and HTS^2^ might be an appropriate approach for anti-metastatic drug discovery.

c-Jun is a protein encoded by the proto-oncogene *JUN*, the cellular homolog of the transforming viral oncogene *v-JUN* in humans. Based on accumulating evidence, c-Jun is involved in a wide variety of cellular processes, including proliferation, differentiation, growth, apoptosis, cell migration and transformation (Lopez-Bergami et al., 2010). c-Jun’s activity is regulated by post-translational modifications that are largely controlled by components of mitogen-activated protein kinase (MAPK) family kinases, including c-Jun N-terminal kinase (JNK), extracellular-signal-regulated kinase (ERK) and p38 kinase. However, few reports have identified a role for c-Jun in breast cancer organ-specific metastasis, especially breast cancer lung metastasis (BCLM).

The aim of the present study was to explore the possibility of using metastasis-associated gene signature-based high-throughput screening to discover anti-BCLM drugs. We used *in vitro* cell migration and colonization assays and *in vivo* mouse models to characterize screening hits. Among thousands of compounds, we determined that Ponatinib, a tyrosine-kinase inhibitor, represses BCLM-associated gene expression via the ERK/c-Jun signaling pathway and inhibits BCLM in mouse models. Our study not only provided new insights into anti-metastatic drug discovery but also revealed c-Jun as a crucial factor and potential drug target for BCLM.

## Results

### Ponatinib is identified by HTS^2^ screening as a potential BCLM-inhibiting compound

**Figure 1:**
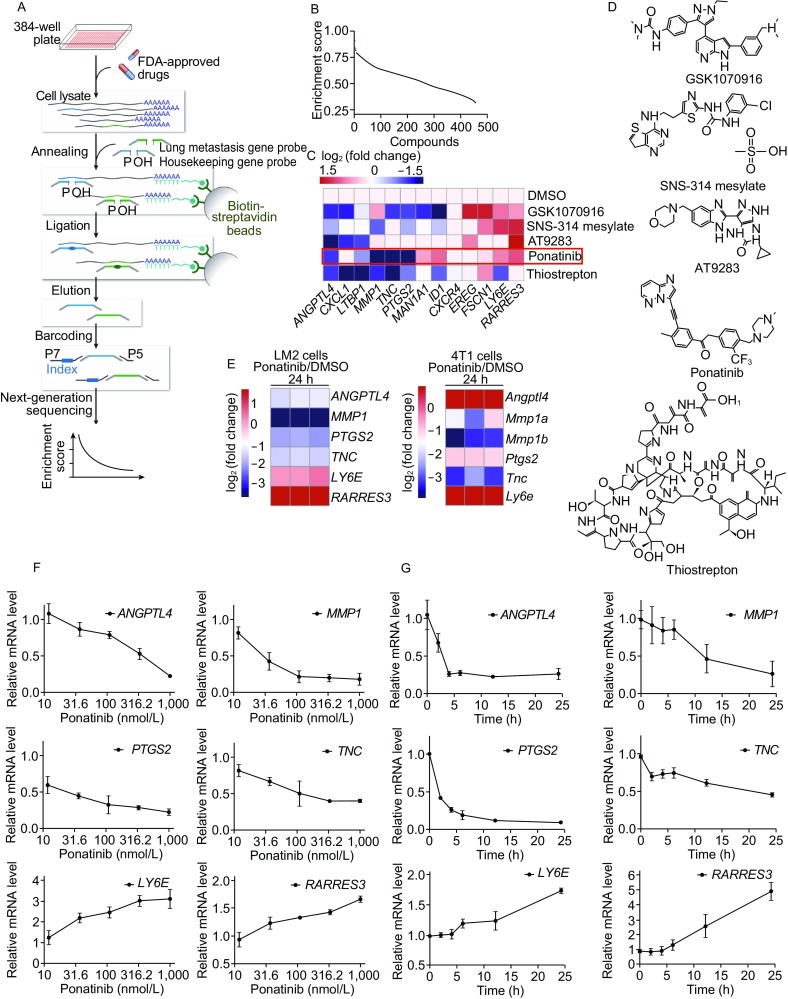
**Ponatinib reverses the expression of certain BCLM-associated genes**. (A) The scheme of the experimental process. (B) Potential anti-metastatic compounds are sorted by the activity score of reversing the BCLM-associated gene signature. The top 5 hit compounds are shown as red dots. (C) Heat map representing the expression of 13 metastatic-related genes (up-regulated, red; down-regulated, blue) in MDA-MB-231 cells treated with the top 5 hit compounds in the screen data, the log_2_ (fold change) is used to scale the differences in gene expression. (D) The top 5 chemical structures are shown. (E) LM2 cells or mouse breast cancer 4T1 cells were treated with 1 µmol/L Ponatinib at 24 h, heat map representing the expression of 6 metastatic-related genes in three independent experiments. (F and G) RT-qPCR analysis showed that Ponatinib was capable of reversing the expression of 6 BCLM-associated genes in a dose- and time-dependent manner. Cells were treated with a serial dilution of Ponatinib at 24 h (F) or with 1 µmol/L Ponatinib at various time points (0, 2, 4, 6, 12 and 24 h) (G). Data present mean ± SD of three independent experiments and normalized to DMSO control

We set up an approach to profile the mRNA levels of 46 genes in the breast cancer MDA-MB-231 cell line using the HTS^2^ method. Among the 46 genes, 13 genes representing the BCLM gene signature (Minn et al., 2005) were chosen from published literature, and 33 genes stably expressed in breast cancer were selected as an internal control (Casey et al., 2009; Clarke et al., 2013). The MDA-MB-231 cell line has been widely used to study breast cancer metastasis and is therefore an appropriate cell model for this screening (Kang et al., 2003; Minn et al., 2005; Bos et al., 2009). The assay had high sensitivity and excellent reproducibility, which were validated in a pilot screen (Fig. S1A and S1B). MDA-MB-231 cells were treated for 24 h with thousands of compounds, including US Food and Drug Administration (FDA)-approved drugs. The activity of each compound was scored according to its ability to reverse gene expression in the BCLM gene signature (Subramanian et al., 2005) (Fig. 1A and 1B). Among the top 5 hit compounds, 2 compounds targeted the aurora kinase, which induce mammary cell migration and breast cancer metastasis via the cofilin-F-actin pathway (Wang et al., 2010). These results demonstrated that our approach can effectively identify potential anti-metastatic drugs. Ponatinib, an FDA-approved drug for leukemia, has not been previously reported to inhibit breast cancer metastasis (O’Hare et al., 2009) (Fig. 1C and 1D). The genes most affected by Ponatinib were chosen to validate the screening results. By RT-qPCR, we confirmed that Ponatinib indeed dose- and time- dependently affected the expression of 6 BCLM-associated genes, including *ANGPTL4*, *MMP1*, *PTGS2*, *TNC*, *LY6E* and *RARRES3* (Fig. 1F and 1G). These genes exhibited similar expression in human breast cancer LM2 cells and mouse breast cancer 4T1 cells (Fig. 1E) when treated with Ponatinib. These findings indicated that the effect of Ponatinib on these 6 BCLM-associated genes may be general. Thus, based on HTS^2^ screening, Ponatinib was selected as an anti-metastatic drug for further study.

### Ponatinib inhibits breast cancer cell migration and colonization *in vitro*

**Figure 2:**
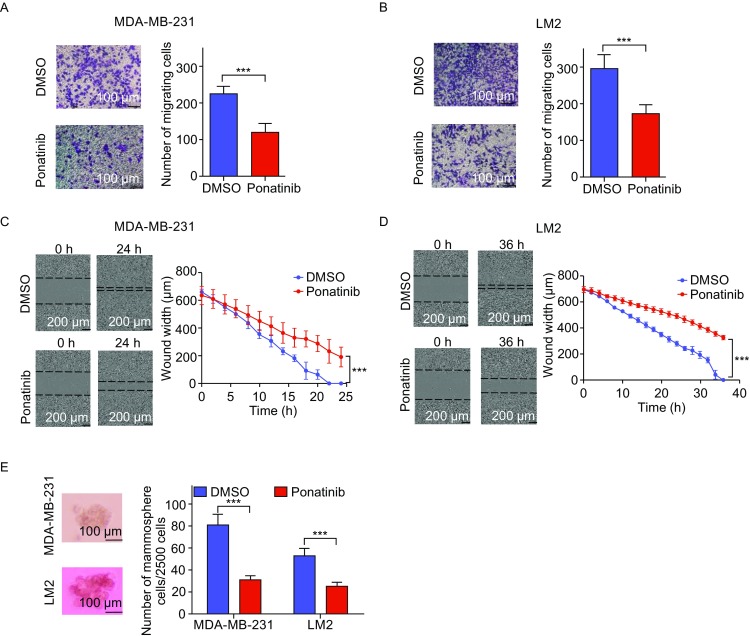
**Ponatinib inhibits breast cancer migration** ***in vitro***. (A and B) The effect of Ponatinib on the migration of MDA-MB-231 cells (A) and LM2 cells (B) was tested by a Transwell migration assay. Cells were treated with 1 µmol/L Ponatinib for 8 h, the migrating cells were stained with crystal violet (left panel) and quantified (right panel). ***P < 0.001 by two-sided Student’s *t*-test. Scale bar, 100 µm. (C and D) The effect of Ponatinib on the migration of MDA-MB-231 cells (C) and LM2 cells (D) was tested by a wound healing assay. Cells were treated with 1 µmol/L Ponatinib, the wound width was photographed after scratching (left panel) and quantified (right panel). *P* value by one-tailed Mann-Whitney *U* test. Scale bar, 200 µm. (E) The effect of Ponatinib on mammosphere formation of MDA-MB-231 cells and LM2 cells was tested by an ultralow attachment microplate. Cells were treated with 1 µmol/L Ponatinib. ***P < 0.001 by two-sided Student’s *t*-test. Scale bars, 100 µm. Representative images of one of three independent experiments are shown. Error bars represent mean ± SD of 6 samples in each group (A–E)

The 4 genes regulated by Ponatinib are involved in cell migration and colonization; thus, we first examined the effect of Ponatinib on cell migration ability. Ponatinib significantly inhibited the migration of MDA-MB-231 cells and LM2 cells (*P* < 0.001) (Fig. 2A and 2B), which was confirmed by another standard assay for testing cell migration, the wound healing assay (*P* < 0.001) (Fig. 2C and 2D). Moreover, mammosphere formation in MDA-MB-231 cells and LM2 cells was significantly inhibited by Ponatinib treatment in an ultralow attachment microplate (Fig. 2E). In addition to the cell migration, our result indicated that Ponatinib could also repress the anchorage-independent growth and colonization, which is also one of the hallmarks of cell transformation.

### Ponatinib inhibits breast cancer metastasis to the lungs *in vivo*

**Figure 3:**
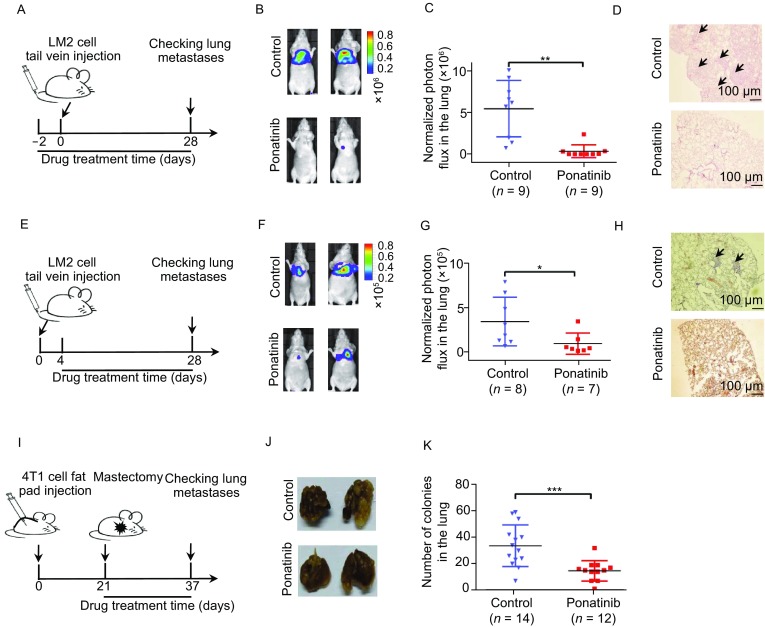
**Ponatinib inhibits breast cancer metastasis to the lung in mouse models**. (A) Schematic representation of mouse experimental design. At day 0, LM2 cells (2 × 10^5^ cells) were injected into the tail veins of mice, and lung colonization was assayed by bioluminescence imaging (BLI). Mice were treated with vehicle or Ponatinib (30 mg/kg/day) for up to 30 consecutive days. (B) Representative bioluminescence images of lung colonization in the mice. (C) Plots show normalized photon flux in the lungs of each group of mice at day 28. *n* = 9 mice per group. **P < 0.01 by two-sided Student’s *t*-test. Error bars, SEM. (D) Representative H&E staining of lung sections are shown. Arrow reflects the metastatic foci detected in lung section. (E) LM2 cells (2 × 10^5^ cells) were injected into the tail veins of mice. After 4 days, the mice received vehicle or Ponatinib (30 mg/kg/day) via oral gavage each day, and lung colonization was quantified. (F) Representative bioluminescence images of lung colonization in the mice. (G) Plots show normalized photon flux in the lungs of each group of mice at day 28. *n* = 8 mice for the control group and *n* = 7 for the Ponatinib treatment group. *P < 0.05 by two-sided Student’s *t*-test. Error bars, SEM. (H) Representative immunohistochemistry images of metastasis cells in mouse lung section. (I) Schematic representation of mouse experimental design. At day 0, 4T1 cells (1 × 10^6^ cells) were injected into the fat pads of mice. When the tumor volume reached 200 mm^3^, the tumors were resected, and the mice were treated with vehicle or Ponatinib (30 mg/kg/day) for up to 16 consecutive days. At day 37, the mice were sacrificed, and metastatic colonies were counted. (J) Representative images of metastases in the lung; arrows indicate metastatic foci. (K) Plots show the metastatic colonies in the lungs of each group of mice. *n* = 14 mice for the control group and *n* = 12 for the drug treatment group. ***P < 0.001 by two-sided Student’s *t*-test. Error bars, SEM

To examine whether Ponatinib effectively prevents breast cancer lung metastasis *in vivo*, we pretreated BALB/c-nude mice with Ponatinib or vehicle at a dose of 30 mg/kg/day through oral gavage for 2 days. Then, LM2 cells, which are derived from MDA-MB-231 cells and show high lung metastatic capability (Minn et al., 2005), were injected into the mouse tail vein, and lung metastatic progression was monitored with bioluminescence imaging (BLI) (Fig. 3A). At 4 weeks post-LM2 cell injection, 8 of 9 mice in the vehicle-treated group presented luciferase activity in the lung, while only 1 of 9 mice in the Ponatinib-treated group exhibited luciferase activity in the lung. Compared with vehicle treatment, Ponatinib treatment inhibited > 90% of cancer cell colonization in the lung as demonstrated by bioluminescence signals (mean 0.35 × 10^6^ vs. 5.46 × 10^6^, *P* = 0.003) (Fig. 3B and 3C) and H&E staining (Fig. 3D).

To further examine whether Ponatinib could inhibit the growth of micro-metastases of breast cancer in mouse lungs, we injected LM2 cells into the tail veins of nude mice and waited for 4 days to let the form of micro-metastases in the lungs (Padua et al., 2008). Then, these mice were treated with Ponatinib or vehicle daily through oral gavage. Lung colonization was examined by lung bioluminescence signals 24 days later (Fig. 3E). Compared with controls, Ponatinib decreased breast cancer cell growth in the lung by as much as 80% according to BLI (mean photon flux of 0.89 × 10^5^ vs. 3.40 × 10^5,^ *P* < 0.05) (Fig. 3F and 3G) and H&E staining (Fig. 3H). Hence, Ponatinib treatment significantly inhibited the growth of micro-metastases in the lungs. To evaluate the general effect of Ponatinib on cancer metastasis, we used a spontaneous metastasis mouse model, which is the gold standard for mimicking the full metastatic process in mice. Mouse breast cancer 4T1 cells were injected into the mammary fat pads of mice to induce primary breast tumor growth. When the volume of primary tumors reached 200 mm^3^, the primary tumors were surgically resected. These mice were randomly grouped and treated with vehicle or Ponatinib by oral gavage. After 16 days of drug treatment, breast cancer metastases were assayed by BLI (Fig. 3I). Quantification of metastatic colonies in the lungs of each mouse established the significant inhibitory effect of Ponatinib on BCLM (mean 16 vs. 33, *P* = 0.0003) (Fig. 3J and 3K).

To investigate whether the inhibition of Ponatinib on breast cancer metastasis was caused by the decreased growth of primary tumor, we orthotopically injected LM2 cells into the mammary fat pads of BALB/c-nude mice, and tumor volume was measured during the three weeks course of drug treatment. The tumor volume at the primary site was not significantly different between mice treated with vehicle control and Ponatinib (*P* = 0.730) (Fig. S2B). Meanwhile, the proliferation of MDA-MB-231 cells and LM2 cells was not sensitive to Ponatinib treatment, the IC_50_ of Ponatinib with these two cells was as high as 1 µmol/L (Fig. S2A). All these data suggested that the anti-metastatic effect of Ponatinib is not due to the growth inhibition of primary tumors *in vivo* and *in vitr*o.

Collectively, supported by two human xenograft models and a spontaneous mouse model, our work clearly indicated that Ponatinib significantly prevents and inhibits breast cancer metastasis to the lungs in mouse models.

### Ponatinib inhibits the expression of BCLM-associated genes only partially via its reported target ABL

**Figure 4:**
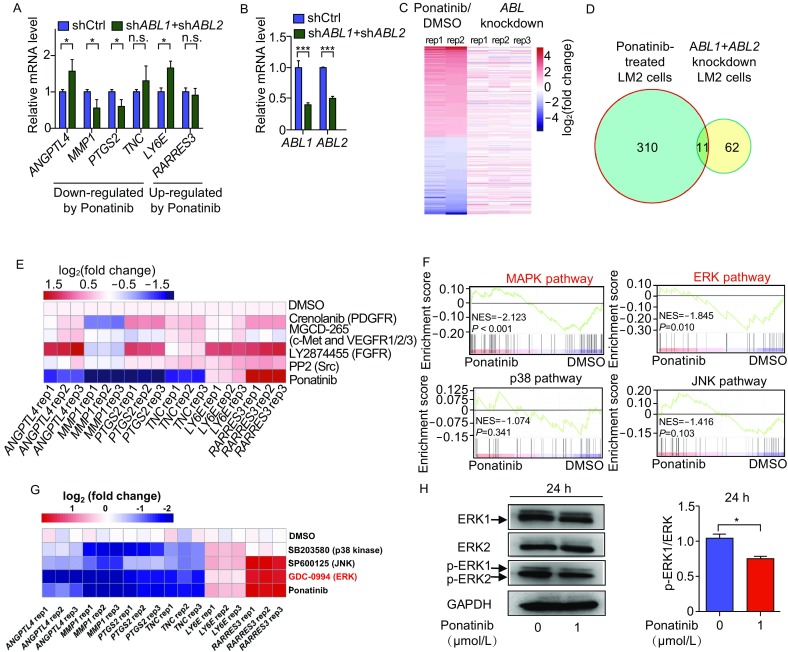
**Ponatinib inhibits the expression of 4 BCLM related genes mainly through the ERK pathway**. (A and B) The 6 BCLM-associated gene expression in control and *ABL1*/*ABL2* double-knockdown LM2 cells is shown in (A), and the knock down efficiency of *ABL1*/*ABL2* is shown in (B). Data are mean ± SD of three independent experiments. *P < 0.05, ***P < 0.001 by two-sided Student’s *t*-test. (C) Heat map of genes differently expressed in Ponatinib-treated LM2 cells, the related genes expression in *ABL1*/*ABL2* or double-knockdown in LM2 cells were also shown. Blue and red colors represent down- and up-regulated genes, respectively. (D) Venn diagrams of overlapped genes with Ponatinib treatment and *ABL1*/*ABL2* double-knockdown in LM2 cells. The differential expressed genes were defined by fold-change > 2 and *P* < 0.05. (E) The common targets of Ponatinib were inhibited with specific inhibitors. LM2 cells were treated with 1 µmol/L Crenolanib (PDGFR inhibitor), 1 µmol/L MGCD-265 (c-Met and VEGFR1/2/3 inhibitor), 1 µmol/L LY2874455 (FGFR inhibitor), 1 µmol/L PP2 (Src inhibitor) individually for 24 h, 6 BCLM-associated gene expression is detected by RT-qPCR. Three independent experiments were performed. (F) GSEA analysis of the indicated gene signatures in DMSO- or Ponatinib-treated cells. NES, normalized enrichment score. *P* value represents false discovery rate adjusted *P*-value. (G) LM2 cells were treated with 20 µmol/L SP600125 (JNK inhibitor), 20 µmol/L SB203580 (p38 MAPK inhibitor), 1 µmol/L GDC-0994 (ERK inhibitor) individually. Gene expression was detected by RT-qPCR. Three independent experiments were performed. (H) LM2 cells were treated with DMSO or 1 µmol/L Ponatinib for 24 h, Western blot analysis of the indicated protein level and phosphorylation level (left panel), related p-ERK level were quantified (right panel). Data represent mean ± SD of three independent experiments. Two-tailed Student’s *t*-test were performed, *P < 0.05

To investigate how Ponatinib affected the expression of BCLM-associated genes, we first examined whether Ponatinib reversed the expression of these 6 genes through ABL, a well-known target of Ponatinib (O’Hare et al., 2009). The *ABL* gene family has 2 members, *ABL1* and *ABL2*. We knocked down the expression of *ABL1* and/or *ABL2* by shRNAs in LM2 cells and measured the expression changes in these BCLM-associated genes by RT-qPCR. The expression of only half of these 6 genes was significantly reversed (Figs. 4A, 4B, S3A and S3B). This result was consistent with a previous report in which the expression of *ABL1* and/or *ABL2* was knocked down in 1833 cells that were also derived from MDA-MB-231 cells; the expression of only 1 of these 6 BCLM-associated genes was reversed (Wang et al., 2016) (Fig. S3C).

To further determine the role of ABL in Ponatinib-mediated inhibition of BCLM, we used RNA sequencing (RNA-seq) to obtain a genome-wide profile of differentially expressed genes (DEGs) in LM2 cells, which were treated with Ponatinib as well as *ABL1* and/or *ABL2* shRNAs (Fig. 4C). As expected, the expression changes in these 6 BCLM-associated genes as well as *ABL1* and *ABL2* genes after Ponatinib treatment or *ABL1* and *ABL2* knockdown were consistent with our RT-qPCR results (Fig. S4A and S4B). RNA-seq also revealed 321 and 73 DEGs (fold change >2, *P* < 0.05) after Ponatinib treatment and *ABL1*/*ABL2* double knockdown, respectively. However, only 15% of *ABL1*/*ABL2* double-knockdown-affected genes were also affected by Ponatinib treatment (11/73 genes) (Fig. 4D). This genome-wide analysis again suggested that ABL might not be the major mediator of Ponatinib’s inhibitory effect on the expression of BCLM-associated genes. Notably, a recent report also mentioned that loss of function of *ABL1* and/or *ABL2* does not inhibit breast cancer metastasis to the lung (Wang et al., 2016), which further demonstrated that *ABL* does not play crucial roles in the anti-metastatic function of Ponatinib. Ponatinib is a multitarget inhibitor of ABL, PDGFRα, VEGFR2, FGFR1 and Src, with a low IC_50_ (O’Hare et al., 2009). Unfortunately, when inhibiting these targets of Ponatinib, we did not identify a target similar to Ponatinib that regulates the expression of these genes (Fig. 4E).

Together, our data clearly showed that ABL plays only a minor role in the regulation of BCLM-associated gene expression by Ponatinib. This result suggested that Ponatinib may regulate BCLM-associated gene expression via unknown or multiple targets.

### Ponatinib inhibits the expression of BCLM-associated genes mainly through the extracellular signal-regulated kinase (ERK) pathway

Because ABL played only a minor role in the inhibitory effect of Ponatinib on metastasis-associated gene expression, we investigated relevant pathways in drug-treated LM2 cells. We first carried out gene ontology (GO) analysis using the Kyoto encyclopedia of genes and genomes (KEGG) database (Subramanian et al., 2005) with RNA-seq data from Ponatinib-treated LM2 cells. Tumor necrosis factor (TNF) and MAPK signaling pathways were mostly enriched (Fig. S5). As a critical cytokine, TNF can induce a wide range of intracellular signaling pathways, and a substantial part of its function is primarily mediated by the MAPK cascade. Therefore, we focused on the MAPK pathway, which was significantly inhibited by Ponatinib treatment in gene-set enrichment analysis (GSEA) (Fig. 4F). ERK, p38 and JNK are the three main molecules downstream of the MAPK pathway (Morrison, 2012). After further GSEA analysis, only the ERK pathway was significantly inhibited by Ponatinib treatment (*P* = 0.01) (Fig. 4F). To further validate this result, we treated LM2 cells with specific inhibitors for the ERK, p38 and JNK pathways and tested the expression of BCLM-associated genes. In RT-qPCR results, the pattern of DEG expression initiated by ERK pathway inhibitor GDC-0994 was indeed more similar to that initiated by Ponatinib treatment but not p38 inhibitor SB203580 or JNK inhibitor SP600125 treatment (Fig. 4G). The phosphorylation of ERK 1/2 was decreased in LM2 cells treated with Ponatinib, consistent with our GSEA analysis (Fig. 4H). Thus, the regulatory effect of Ponatinib on BCLM-associated genes might be mainly carried out via the ERK pathway.

### Transcription factor c-Jun, which directly binds to and activates BCLM-associated genes, is inhibited by Ponatinib

To understand how these BCLM-associated genes are regulated by Ponatinib treatment, we examined the expression of these 6 BCLM-associated genes in detail. Four genes (*ANGPTL4*, *MMP1*, *PTGS2* and *TNC*) and 2 genes (*LY6E* and *RARRES3*) from the BCLM gene signature were inhibited and activated by Ponatinib treatment, respectively. This observation suggested that at least two different regulatory mechanisms are involved in the reversal of BCLM-associated gene expression by Ponatinib.

**Figure 5:**
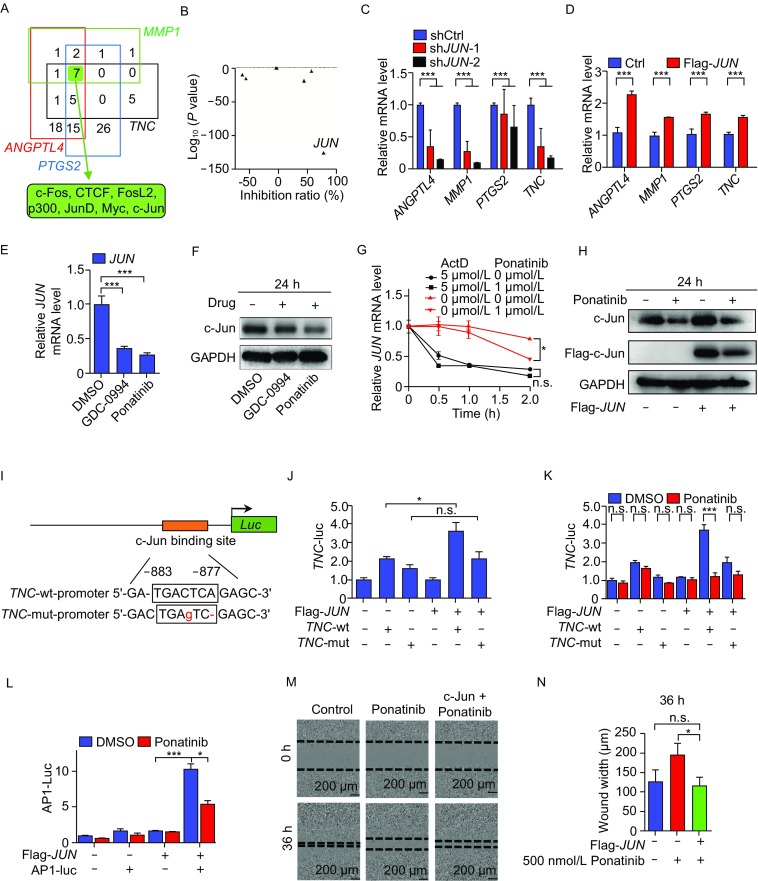
**Ponatinib inhibits the expression of certain BCLM-associated genes and cell migration via the transcription factor c-Jun**. (A) Transcription factors that bind to the promoters of 4 BCLM-associated genes down-regulated by Ponatinib in ENCODE ChIP-seq data. The numbers in each color box represent the number of transcription factors bound to the promoter region of each BCLM related genes (group), the shared 7 transcription factors in four groups were marked with a green solid box. (B) Gene expression analysis of these 7 transcription factors in (A) when LM2 cells were treated with Ponatinib. (C and D) RT-qPCR analysis of the expression of 4 BCLM-associated genes in LM2 cells with knockdown (C) or overexpression (D) of *JUN*. (E) RT-qPCR analysis of *JUN* mRNA expression in LM2 cells treated with 1 µmol/L Ponatinib or 10 µmol/L GDC-0994 for 24 h. (F) Western blot analysis of the protein level of endogenous c-Jun in LM2 cells treated with 1 µmol/L Ponatinib or 10 µmol/L GDC-0994 for 24 h. (G) LM2 cells were treated with 1 µmol/L Ponatinib and 5 µmol/L actD for the indicated time, RT-qPCR was used to analyze the *JUN* mRNA expression. (H) LM2 cell were transfected with c-Jun or empty vector for 48 h, then treated with DMSO or 1 µmol/L Ponatinib for 24 h, Western blot analysis of the protein level of endogenous or transfected c-Jun. (I) Schematic representation of the location of the c-Jun DNA binding site (AP1) in the *TNC* promoter. (J and K) LM2 cells were transfected by luciferase reporter vectors driven by *TNC* wild-type or mutant promoters and *JUN* overexpression or control vectors. Next, luciferase activity was determined (J); or transfected cells were treated with DMSO or 1 µmol/L Ponatinib for 24 h, then luciferase activity was examined (K). (L) Suppression of AP1 site-Luc activity by Ponatinib in c-Jun-overexpressing LM2 cells. (M and N) MDA-MB-231 cells were transfected with c-Jun or empty vector for 48 h, and treated with DMSO or 500 nmol/L Ponatinib. Cells were scratched and the wound width was photographed. Representative images of one of three independent experiments are shown (M) and quantified (N) among the three groups at 36 h after Ponatinib treatment. Data represent mean ± SD of three independent experiments in (C–H, J–L and N). Two-tailed Student’s *t*-test were performed in (C–E, J–L and N), ***P < 0.001, **P < 0.01, *P < 0.05

To illustrate how these 4 genes are down-regulated by Ponatinib treatment, we examined which transcription factors (TFs) bind the promoters of these 4 genes. After checking ENCODE ChIP-seq data (Raney et al., 2011), we identified 7 TFs with known putative binding sites for all 4 promoters (Fig. 5A). Analysis of RNA-seq data from Ponatinib-treated LM2 cells revealed that among these 7 TFs, only *JUN* expression was significantly down-regulated (Fig. 5B), which indicated that c-Jun might contribute to the regulation of these 4 genes by Ponatinib. Next, we knocked down *JUN* mRNA by 2 shRNAs with different sequences in LM2 cells and found that the expression of 3 genes was indeed down-regulated (Figs. 5C and S6A). Moreover, overexpression of *JUN* led to significant activation of the expression of these 4 genes (Figs. 5D and S6B). In addition, the expression of *JUN* and these 4 BCLM-associated genes was higher in lung metastatic LM2 cells than in parental MDA-MB-231 cells (Lu et al., 2010), which indicates that c-Jun may play a role in the process of BCLM. Collectively, our results suggested that c-Jun activates the expression of these 4 BCLM-associated genes.

The repression of Ponatinib treatment on the expression of *JUN* was confirmed by RT-qPCR analysis on mRNA level and Western blot analysis on protein level. As expected, the inhibition of ERK by compound GDC-0994 on the expression of *JUN* shows the similar trend (Fig. 5E and 5F). To determine how Ponatinib regulates the expression of *JUN*, we treated LM2 cells with actinomycin D (actD) as well as Ponatinib. ActD is an antibiotic that inhibits DNA-primed RNA polymerase and is widely used as a transcription inhibitor. Our data indicated that Ponatinib significantly inhibited the transcription of *JUN* (*P* < 0.05) but had no effect on the stability of *JUN* mRNA (Fig. 5G). Western blot results showed that Ponatinib treatment accelerated the degradation of c-Jun protein, regardless of whether it was translated from the endogenous or transfected *JUN* gene (Fig. 5H). These results suggested that Ponatinib inhibits the expression of *JUN* in mRNA and protein levels.

To assess whether c-Jun directly binds to and regulates the expression of these 4 genes, we chose the *TNC* promoter as an example for a reporter assay. c-Jun regulates the responsive promoter via binding to its canonical TGAG/CTCA motif (AP1) (Angel et al., 1987). We identified one potential c-Jun binding site at −883 to −877 bp upstream of the *TNC* transcription start site (TSS) (Fig. 5I). To determine whether the putative c-Jun binding site is involved in *TNC* transcription, we constructed reporter vectors with the DNA sequence of the *TNC* promoter containing a wild-type (*TNC*-wt) or mutated (*TNC*-mut) putative c-Jun binding site (Fig. 5I). These constructs were co-transfected into LM2 cells with or without a *JUN* expression plasmid. *JUN* overexpression activated luciferase gene expression, which was driven by the *TNC*-wt promoter but not the *TNC-*mut promoter (*P* < 0.05 and *P* > 0.05, respectively) (Fig. 5J). Notably, the luciferase gene expression drove by *TNC*-wt promoter and c-Jun overexpression was diminished significantly by Ponatinib treatment (*P* < 0.001) (Fig. 5K).

To determine whether Ponatinib treatment inhibits the expression of *TNC* only or c-Jun target genes in general, we transfected LM2 cells with a reporter vector containing 3 × AP1 sites combined with the luciferase gene with or without a *JUN* expression plasmid. The overexpression of *JUN* significantly activated luciferase gene expression (*P* < 0.001), and this activation was significantly inhibited by Ponatinib treatment (*P =* 0.011) (Fig. 5L). These results clearly indicated that Ponatinib treatment can effectively inhibit the expression of c-Jun target genes in general.

Together, our findings suggested that Ponatinib treatment represses the expression of c-Jun through inhibiting its transcription as well as accelerating c-Jun protein degradation, which leads to less binding of c-Jun to the promoters of BCLM-associated genes and consequently inhibits the transcription of these genes.

### c-Jun rescues the migration of breast cancer cells treated with Ponatinib

To examine whether c-Jun is functionally involved in the migration of breast cancer cells, a wound healing assay was performed. The migration of LM2 cells was significantly inhibited by knocking down *JUN* expression (Fig. S7A and S7B). This result was consistent with a previous report that c-Jun is associated with cell movement in other breast cancer cell lines (Chen et al., 2009). In addition, overexpressed *JUN* partially rescued the migration of breast cancer cells treated with Ponatinib (*P <* 0.001), which indicated that c-Jun might be a major mediator of Ponatinib’s inhibitory effect on cancer cell migration (Figs. 5M, 5N, S6C and S8A). This result was also confirmed by an alternative method Transwell assay, which is also popular for testing cell migration (*P* < 0.01) (Fig. S8B and S8C)

### The expression of *JUN* is positively correlated with the expression of four BCLM-associated genes in breast cancer tumors

**Figure 6:**
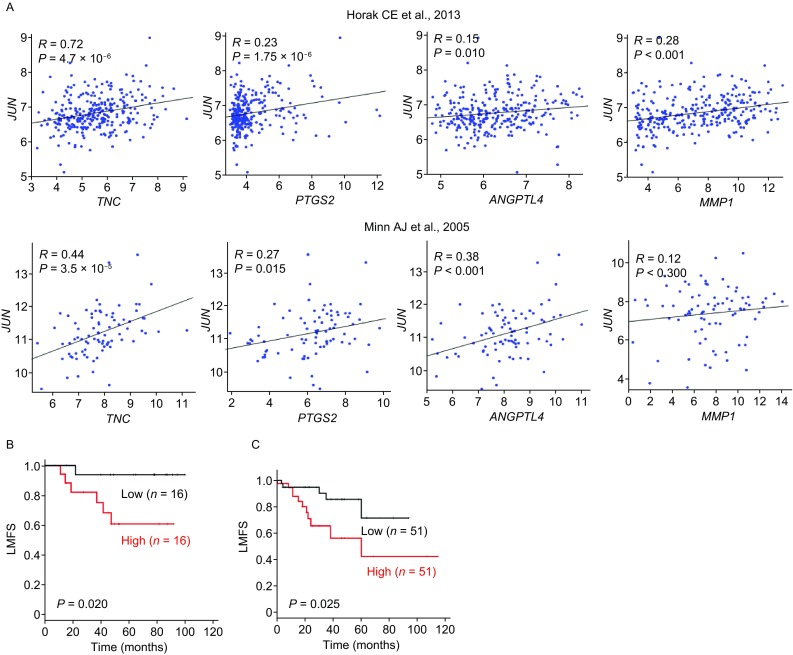
**Increased expression of** ***JUN*** **is correlated with BCLM-associated gene expression and BCLM**. (A) Positive correlation between the expression of *c-Jun* and 4 BCLM-associated genes from 2 independently published datasets GSE41998 (upper panel) and GSE2603 (lower panel). Each spot represents a tumor sample. (B and C) Kaplan-Meier analysis of the probability of cumulative lung metastasis-free survival (LMFS) in 82 and 204 breast cancer cases GSE2603 (B) and GSE12276 (C) by low and high *JUN* expression. *P* values were calculated by log-rank test

To evaluate whether the regulation of *c-Jun* on these 4 BCLM-associated genes also occurs in breast cancer patients, we analyzed the transcriptome of 279 breast tumors from a previous publication (GSE41998) (Horak et al., 2013) and found that the expression of *JUN* was significantly and positively correlated with the expression of the 4 genes (Fig. 6A, upper panel). Notably, the expression of these 4 genes are also positively correlated with the expression of *JUN* in another dataset, and the correlation from 3 of these 4 genes is significant (*P* < 0.05), except *MMP1* (*P* < 0.300) (GSE2603) (Minn et al., 2005) (Fig. 6A, lower panel).

### Increased expression of *JUN* is associated with the incidence of BCLM

To determine whether *JUN* expression is associated with BCLM we analyzed the correlation of *JUN* expression in primary breast tumors with patient prognosis. We examined the association between *JUN* mRNA levels and organ-specific metastasis within 82 tumor samples based on a published dataset (GSE2603) (Minn et al., 2005). Compared with patients with low expression of *JUN*, patients with high expression of *JUN* exhibited significantly high chance of lung metastasis (*P* = 0.02) (Fig. 6B). Furthermore, this result could also be confirmed in other 204 tumor samples from another published dataset (GSE12276) (Bos et al., 2009) (*P* = 0.025) (Fig. 6C). Together, our findings from clinical samples demonstrated a positive association between the expression of *JUN* and lung metastasis in patients with breast cancer, and suggested that c-Jun play an important role in BCLM.

## Discussion

**Figure 7:**
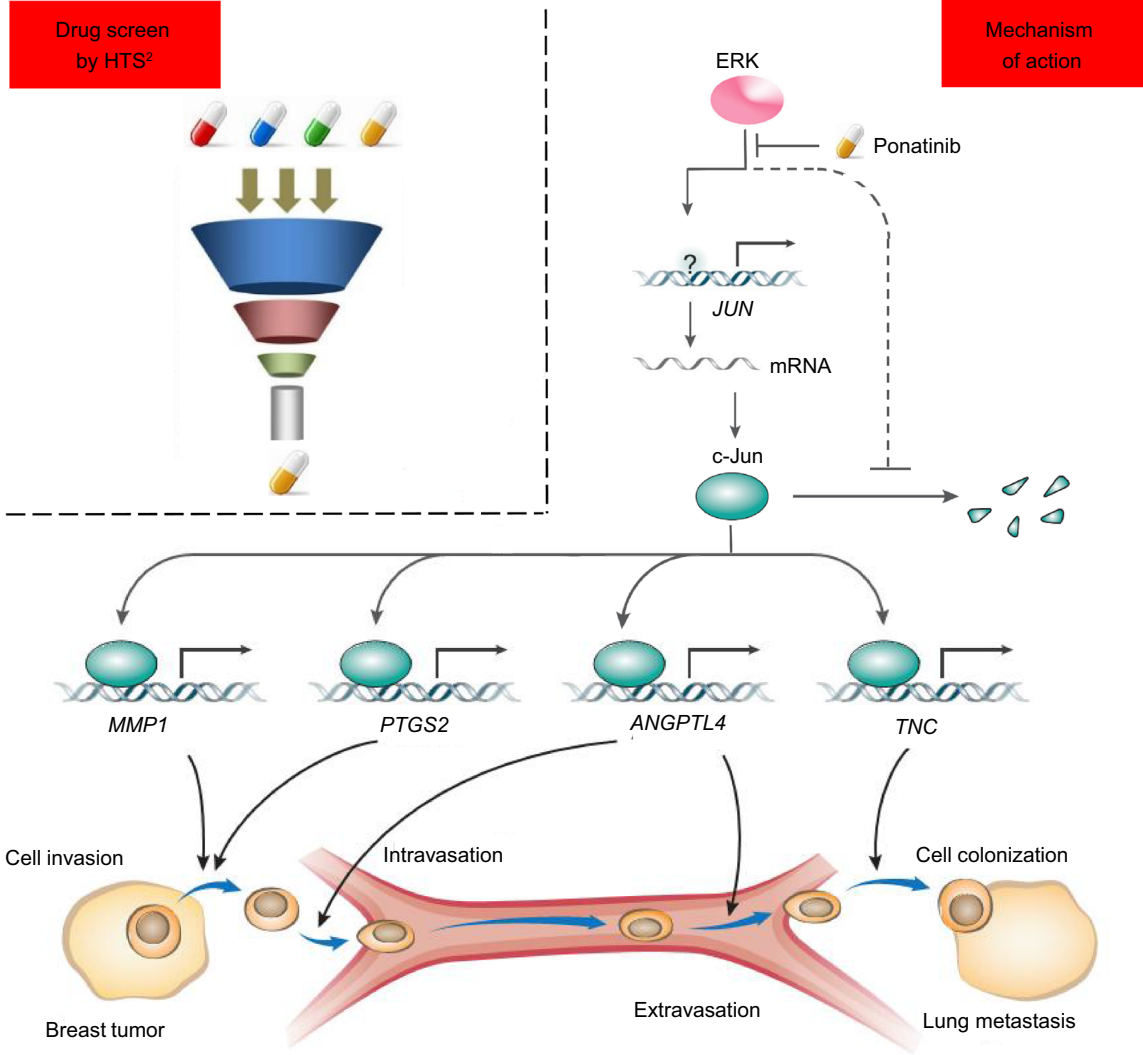
**Proposed model for the role of inhibition of BCLM by Ponatinib**. By using gene signature based drug screen strategy, a series of potential anti-breast cancer lung metastasis (BCLM) drugs were found. Among these drugs, Ponatinib inhibits c-Jun function by repressing mRNA transcription and accelerating degradation of c-Jun protein via ERK/c-Jun signal pathway. Through c-Jun, Ponatinib down regulates 4 BCLM related gene expression, finally inhibits BCLM in mouse models

Approximately 90% of cancer patients die from metastasis (Weigelt et al., 2005), the discovery of anti-metastatic drugs is extremely crucial for the therapeutic treatment of patients with late stage cancers. In this study, to address this critical problem, we used a novel approach by combining HTS^2^ and metastasis-associated genes for high-throughput screening of anti-metastatic drugs. Notably, the expression of metastasis-associated genes was utilized as a readout of drug screening by HTS^2^. Using this new approach, we determined that Ponatinib, which can reverse the expression of 6 BCLM-associated genes, inhibits the lung metastasis of breast cancer. Mechanistically, this anti-metastatic function of Ponatinib is mainly acted through inhibition of the expression of transcription factor c-Jun but not ABL (Fig. 7).

To date, the cell phenotype-based screening is a popular approach for drug discovery and contributes significantly for the development of FDA-approved drugs. However, cell phenotype-based screening has drawbacks, include: 1) the requirement of engineering a separate reporter cell line, 2) most screens depend on a single or a limited number of surrogate readouts, 3) toxic components are hard to filter out, 4) many diseases lack good “drug” targets for developing an effective screening strategy. Gene signature based screen could overcome these drawbacks effectively. 1) Because detection of gene expression, engineering cell model is unnecessary. 2) Through analyzing the housekeeping and cytotoxicity-related gene perturbations by drugs, the toxic compounds could be filtered at the early stages of screening. 3) In addition, known drug targets are not needed for drug development. 4) Importantly, HTS^2^ takes advantage of powerful next-generation sequencing technologies and greatly enhances the parallel processing of samples and genes. These advantages help gene signature based approach to be complementary to cell phenotype-oriented screening in drug discovery.

There have been a number of applications of this approach with a particular focus on drug discovery, such as identifying new therapeutics (Lee et al., 2016), revealing mode of actions for existing chemicals (Saito et al., 2009). For anti-metastatic drug discovery, more works are needed, such as setting up unbiased gene expression signature from publications by other lab (Landemaine et al., 2008; Harrell et al., 2012), or using this approach to other types of organ specific cancer metastasis.

Ponatinib is developed to treat chronic myeloid leukemia (CML) and Philadelphia- chromosome positive (Ph+) acute lymphoblastic leukemia (ALL) with Bcr-Abl T315I mutation. The present findings proved that Ponatinib could prevent and inhibit breast cancer lung metastasis in mouse models, may provide a possibility of new perspectives for this drug. More work is needed to identify the direct target(s) of Ponatinib, which could regulate the expression of c-Jun in breast cancer cells.

In the present study, we demonstrated that c-Jun directly binds to and activates at least 4 BCLM-associated genes, including *ANGPTL4*, *MMP1*, *PTGS2* and *TNC*. These 4 genes contribute to multiple steps of the metastatic process, such as endothelial tight junction (Le Jan et al., 2003), cell migration and invasion (Birkedal-Hansen et al., 1993; Howe et al., 2005), cell differentiation and colonization (Oskarsson et al., 2011). Loss of the function of any of these 4 genes could significantly impair breast cancer metastasis *in vitro* and *in vivo* (Gupta et al., 2007). Since c-Jun can activate the expression of all of these 4 genes, which strongly suggests that c-Jun might be a key factor in the process of breast cancer cells metastasizing to the lung and a promising drug target for anti-metastasis.

In summary, in this study, we established a novel approach for the discovery of anti-metastasis drugs, identified Ponatinib as a new and effective drug to inhibit breast cancer lung metastasis in mouse models and revealed c-Jun as a crucial factor and potential drug target for BCLM. Our current studies may pave the way to drug discovery and therapeutic treatment for metastasis, the leading cause of cancer patient mortality.

## Materials and Methods

### Chemicals

The screening library (1,154 FDA approved drugs), GDC-0994 (S7554), Crenolanib (CP-868596), MGCD-265 (S1361), LY2874455 (S7057), PP2 (S7008), GDC-0994 (S7554) and Ponatinib (S1490) were purchased from Selleck. SP600125 (HY-12041), SB203580 (HY-10256) were purchased from MedChem Express. Actinomycin D (A4262), B27 (17504044) and EGF (100-47) were purchased separately from Sigma, Invitrogen and Peprotech. All chemicals were dissolved in dimethyl sulfoxide (DMSO) for *in vitro* studies.

### Antibodies and plasmids

c-Jun antibody (#9165) and GAPDH antibody (#2118) were purchased from Cell Signaling, anti-Flag antibody (F1804) was purchased from Sigma-Aldrich. For gene knockdown, the shRNA sequence for scrambled shRNA is GGTGTATGGGCTACTATAGAA, others plasmids were purchased from sigma including *ABL1* (TRCN0000039898), *ABL2* (TRCN0000218815) and *JUN* (TRCN0000039590 and TRCN0000039591). To construct the human *JUN* ectopic expression plasmid, pcDNA3.1-*JUN*, a full-length human *JUN* cDNA was cloned from LM2 cDNA. pGL3-AP1-Luc containing 3× c-Jun binding motif (AP1) was purchased from addgene. The *TNC* promoter sequence (−1054 to +246 bp related to TSS) was cloned from DNA of LM2 cells, and inserted into the pGL3-vector. To generate the *TNC*-mutation promoter, multiple site-directed mutagenesis was used to introduce mutations into wild-type *TNC* promoter.

### Cell culture

MDA-MB-231 and 293T cells were obtained from China Infrastructure of Cell Line Resources (Beijing, China), LM2 (Luc+) cells and 4T1 (Luc+) cells were from Dr. Joan Massague (Memorial Sloan Kettering Institute, NY). MDA-MB-231 and 4T1 (Luc+) cells were cultured in RPMI1640 medium (Gibco) containing 10% fetal bovine serum (Gemini) and 100 units/mL streptomycin and penicillin (Gibco), LM2 and 293T cells were maintained in DMEM medium (Gibco) supplemented with 10% FBS and 100 units/mL PS. All the mentioned cell lines were incubated in a 5% CO_2_ atmosphere at 37 °C. All cell lines were authenticated by using PCR for short tandem repeats and verified to be free of Mycoplasma.

### Drug screening

About 3,000 MDA-MB-231 cells were seeded per well in 384-well plates. At 24 h, cells were treated with the small molecules from screening library at 1 µmol/L drugs for 24 h. HTS^2^ assay was performed to quantify the mRNA level of the target gene as described (Li et al., 2012a). The cells were lysed in GentLys buffer (Nanopure, China).

### Screening data processing

Reads were mapped to the probe sequences allowed 3 mismatches, and were normalized by using the expression of 33 stable genes in breast cancer (GSE42568, GSE10797). For each compound treatment, fold-change of gene expression was computed with the normalized signal of drug treatment and averaged normalized signal of DMSO treatment in the same 384-well plate. Genes with fold change >2 and *P* < 0.05 were considered differently expressed genes (DEGs). The method of calculating the anti-metastatic activity score of each compound was similar with described previously (Subramanian et al., 2005).

### Real-time quantitative PCR

Total RNA was extracted by using TRIzol reagent (Invitrogen). Full-length cDNA was synthesized by using the RevertAid first strand cDNA synthesis kit (Thermo). Real-time quantitative PCR were performed by using the KAPA SYBR FAST qPCR Kit (Kapa Biosystems). The gene specific primers are in Table S1.

### Transwell migration assay

The assay was based on the published protocol (Li et al., 2016). Briefly, MDA-MB-231 cells or LM2 cells were seeded in the upper chamber of the Transwell insert (Corning) with serum-free medium. 1 µmol/L Ponatinib were added into the chamber. After treatment for 8 h, migrated cells on the lower membrane were fixed with 4% paraformaldehyde, stained with 0.5% crystal violet and counted by using Image J. For rescue experiment, LM2 cells were transfected with c-Jun or empty vector for 48 h, and treated with DMSO or 500 nmol/L Ponatinib, and then the transwell migration assay was performed.

### Wound healing assay

The assay was based on the published protocol (Thakur et al., 2015). Briefly, MDA-MB-231 cells or LM2 cells at 80%–90% confluence were seeded in a 96-well plate. The wound was made through the cell layer by using a pin tool. After washing with phosphate buffered saline (PBS), complete media was added with or without 1 µmol/L Ponatinib. The wound width was photographed under a light microscope. The kinetic curve of wound width was used to indicate the cell motility potential. For rescue experiment, MDA-MB-231 cells were transfected with c-Jun or empty vector for 48 h, and treated with DMSO or 500 nmol/L Ponatinib, and then the wound healing assay was performed.

### Mammosphere formation assay

The assay was based on the published protocol (Thakur et al., 2015). 1,000 cells (MDA-MB-231, LM2) were seeded in ultra-low attachment microplates (Corning). Cells were grown in a serum-free medium supplemented with 2% B27 (Invitrogen), 20 ng/mL EGF (Peprotech), 1% PS and 1 µmol/L Ponatinib. Media was changed every 3 day for 2 weeks and mammospheres were counted.

### Animal studies

BALB/c-nude female mice 5–6 weeks old were used for xenograft experiments. Experimental metastasis assay was performed as described previously (Minn et al., 2005; Padua et al., 2008). Briefly, 2 × 10^5^ LM2 cells were washed and harvested in PBS and then injected into the lateral tail vein of mouse in a volume of 0.1 mL (day 0). Lung metastasis was detected by bioluminescence imaging (BLI) with an IVIS Lumina II instrument (PerkinElmer). Mice underwent oral gavage with Ponatinib (30 mg/kg/day) or vehicle (25 mmol/L citrate buffer, pH 2.75) (O’Hare et al., 2009). For orthotopic metastasis assay, 1 × 10^6^ 4T1 cells were injected in 50 µL Matrigel (Corning) and diluted with PBS (1:1), then the cells were injected into the mammary fat pad of BALB/c female mice. The mammary tumor growth was regular measurement using a digital caliper. When the tumor volume reached 200 mm^3^, tumors were resected and mice were treated once daily by oral gavage with vehicle (25 mmol/L citrate buffer, pH 2.75), or Ponatinib (30 mg/kg/day) for up to 16 consecutive days. At 37 days, mice were sacrificed. The lungs were fixed in 4% paraformaldehyde and statistics for the metastatic colony. For the primary tumor growth model, BALB/c-nude female mice were orthotopically injected with 1 × 10^6^ LM2 cells into the mammary fat pad. Tumors formed by LM2 cells were measured with digital calipers and tumor volume was calculated with the formula: volume (mm^3^) =  [width^2^ (mm^2^) ×  length (mm)]/2.

### RNA-sequencing (RNA-seq) analysis

LM2 cells at 3 × 10^6^ were plated in a 10-cm petri dish for 24 h then treated with Ponatinib at 1 µmol/L for 6 h. Cells were harvested and RNA was isolated by using TRIzon (Invitrogen). The libraries were constructed by using the NEBNext Ultra™ RNA Library Prep Kit for Illumina (NEB) and sequenced on HiSeq 2500 sequencing system (Illumina). RNA-seq data were mapped to the reference genome (hg38) by using Bowtie/TopHat. The reads were counted, and the differential expression between experimental groups was quantified by using edgeR (Fold change > 2 and *P* value < 0.05).

### Gene ontology (GO) and gene-set enrichment analysis (GSEA) analysis

GO analysis was performed in DAVID (https://david.ncifcrf.gov/) with 321 DEGs (fold change > 27 and *P* value < 0.05) by using the KEGG database. For GSEA, we used a ranked gene list by decreasing fold-change expression of genes. The gene signatures of the MAPK, ERK, p38 and JNK pathway were from the BioCarta pathway and canonical pathway database. Genes were aligned to the ranked list and the running sum was calculated and lead to an enrichment score. The *P* values were calculated on the basis of random permutations.

### Viral transduction

For gene knockdown, a lentiviral vector containing shRNA together with psPAX2 packaging and the pMD2.G envelope plasmid were co-transfected into 293T cells at the ratio of 4:3:1 (PLKO.1:psPAX2:pMD2.G) by using DNAFect Transfection Reagent (CW Biotech, Beijing, China). At 48 h, culture media were harvested, filtered and stored at −80 °C. Viral particles were incubated with cells for 48 h, and surviving cells were harvested after selection with puromycin.

### Transient transfection

For gene-overexpression experiments, 2 × 10^5^ cells were transfected with pcDNA3.1 (vector) or pcDNA3.1-*JUN* by using DNAFect Transfection Reagent. At 24 h after transfection, cells underwent RT-qPCR and Western blot to verify transfection efficiency.

### Western blot

Cells were harvested by RIPA lysis buffer containing 1 mmol/L PMSF and 1× cocktail (Biotool, Beijing). By BCA protein quantification (Pierce), protein samples were separated by SDA-PAGE gel and transferred to nitrocellulose membranes (Amersham Pharmacia), and probed with the indicated antibodies. The bands were visualized by using Pierce ECL Western blot substrate (Amersham). GAPDH was an internal control.

### Actinomycin D assay

Actinomycin D (actD) assay was performed as described previously (Xiu et al., 2017). Briefly, cells were treated with 1 µmol/L Ponatinib and actD at 5 µmol/L for 0, 0.5, 1 or 2 h prior to RNA extraction with TRIzol reagent. Subsequently, RT-qPCR was used to analyze changes in RNA levels.

### Luciferase reporter assay

Luciferase assay was performed as described previously (Wu et al., 2010). Briefly, LM2 cells were seeded in 24-well plates in triplicate 1 day before transfection. An amount of 750 ng reporter vector of the pGL3-wt/mut-*TNC* promoter or empty vector, 150 ng of the internal control plasmid pRL-TK and 750 ng plasmid for the expression of pcDNA3.1-*JUN* or empty vector was co-transfected as indicated per well. At 24 h after transfection, reporter activity of samples was assayed by using the Dual Luciferase Assay System (Promega). To assess whether the Ponatinib worked through c-Jun regulated expression of *TNC*, transfected samples were treated with DMSO or Ponatinib for 24 h, then reporter activity was detected by the same method. The pGL3-AP1-Luc reporter assay was performed similar with *TNC* reporter assay, LM2 cells were co-transfected with pGL3-AP1-Luc or empty vector, pRL-TK and pcDNA3.1-*JUN* or empty vector for 48 h, and treated with DMSO or Ponatinib for 24 h, and then dual luciferase assay was performed.

### Correlation analysis of *JUN* with four BCLM related genes

For the correlation analysis of *JUN* with BCLM-related genes including *ANGPTL4*, *MMP1*, *PTGS2* and *TNC* in breast cancer samples, datasets GSE41998 (*n* = 279) and GSE2603 (*n* = 82) were used. Pearson correlation coefficient was used for correlation analysis.

### Clinical data analysis

For survival analysis of the relationship of *JUN* expression with the breast cancer metastasis organ, the levels of *JUN* and clinical metastasis data were acquired from the datasets GSE2603 (*n* = 82) and GSE12276 (*n* = 204) and the original reports. The ranked expression levels were classified into the top 20/25%, bottom 20/25% and middle 60/50%, respectively, then Kaplan-Meier survival curves were drawn and log-rank test *P* values were calculated by using the R package “survival”.

### Statistical analysis

Comparisons of two groups involved two-tailed unpaired Student’s *t*-test, log-rank test was used for Kaplan-Meier survival analysis, Pearson’s product-moment correlation test was used for gene correlation analysis, and one-tailed Mann-Whitney test was used for wound healing test. The level of significance was set at *P < 0.05, **P < 0.01, ***P < 0.001.

### Data availability

RNA-seq data have been deposited in the gene expression omnibus (GEO) database under access number GSE95079. All relevant data are available from the authors on request.

## Electronic supplementary material

Below is the link to the electronic supplementary material.

Supplementary material 1 (PDF 243 kb)

Supplementary material 1 (PPTX 1,860 kb)
