# Supplementary material for: Chemical genomics reveals inhibition of breast cancer lung metastasis by Ponatinib via c-Jun
Source: Protein Cell. 2018 Apr 17;10(3):161–77. doi: 10.1007/s13238-018-0533-8 (PMC6338618; doi:10.1007/s13238-018-0533-8)
Supplement: Supplementary file 1 — Supplementary material 1 (PDF 243 kb) [file 13238_2018_533_MOESM1_ESM.pdf]

Supplementary Table 1. List of primer sequences used in this study.

F: forward primer; R: reverse primer.

Primer names	Primer sequences (5' – 3')
qPCR-human-*ANGPTL4*-F	GATGGCTCAGTGGACTTCAACC
qPCR-human-*ANGPTL4*-R	TGCTATGCACCTTCTCCAGACC
qPCR-human-*MMP1*-F	ATGAAGCAGCCCAGATGTGGAG
qPCR-human-*MMP1*-R	TGGTCCACATCTGCTCTTGGCA
qPCR-human-*PTGS2*-F	CGGTGAAACTCTGGCTAGACAG
qPCR-human-*PTGS2*-R	GCAAACCGTAGATGCTCAGGGA
qPCR-human-*TNC*-F	ATGTCCTCCTGACAGCCGAGAA
qPCR-human-*TNC*-R	AGTCACGGTGAGGTTTTCCAGC
qPCR-human-*LY6E*-F	GACCAGGACAACTACTGCGTGA
qPCR-human-*LY6E*-R	AAGCCACACCAACATTGACGCC
qPCR-human-*RARRES3*-F	GCAGGAACTGTGAGCACTTTGTC
qPCR-human-*RARRES3*-R	GCAACAACCAGGATTCCAAGCG
qPCR-human-*ATCB*-F	CACCATTGGCAATGAGCGGTTC
qPCR-human-*ATCB*-R	AGGTCTTTGCGGATGTCCACGT
qPCR-mouse-*ANGPTL4*-F	CTGGACAGTGATTCAGAGACGC
qPCR-mouse-*ANGPTL4*-R	GATGCTGTGCATCTTTTCCAGGC
qPCR-mouse-*MMP1A*-F	AGGAAGGCGATATTGTGCTCTCC
qPCR-mouse-*MMP1A*-R	TGGCTGGAAAGTGTGAGCAAGC
qPCR-mouse-*MMP1B*-F	GCAGTTGTGGAAGATGCCATCG
qPCR-mouse-*MMP1B*-R	CCATCAAATGTGTAGAGGTCACC
qPCR-mouse-*PTGS2*-F	GCGACATACTCAAGCAGGAGCA
qPCR-mouse-*PTGS2*-R	AGTGGTAACCGCTCAGGTGTTG
qPCR-mouse-*TNC*-F	GAGACCTGACACGGAGTATGAG
qPCR-mouse-*TNC*-R	CTCCAAGGTGATGCTGTTGTCTG
qPCR-mouse-*LY6E*-F	CCTGATGTGCTTCTCATGTACCG
qPCR-mouse-*LY6E*-R	GTTCAGGGTGTAGCCAAGGTTG
qPCR-mouse-*ACTB*-F	CATTGCTGACAGGATGCAGAAGG
qPCR-mouse-*ACTB*-F	TGCTGGAAGGTGGACAGTGAGG
qPCR-human-*ABL1*-F	GGCTGTGAGTACCTTGCTGC
qPCR-human-*ABL1*-R	GGCGCTCATCTTCATTCAGGC
qPCR-human-*ABL2*-F	CCAGCTACTCCCGAGGCTG
qPCR-human-*ABL2*-R	CTTGATCCCACAGGGTGAAG
qPCR-human-*JUN*-F	CAACATGCTCAGGGAACAGG

qPCR-human-*JUN*-R	GTTAGCATGAGTTGGCACCC
3.1-*JUN*-F	CTAGCTAGCGCCGCGCCACCATGGAAACTGCAAAGATGGAAACGAC
3.1-*JUN*-R	CTAGTCTAGATCAAAATGTTTGCAACTGCTGCGTTA

Figure S1. Validation of the HTS^2 platform. (A) Validation of HTS^2 sensitivity. We performed a HTS^2 assay with 3,000 and 5,000 MDA-MB-231 cells, and PCR products were observed by agarose gel electrophoresis. (B) Pearson's correlation matrix of repeat samples in inter-plates and intro-plates. Gene expression data (HTS^2 normalized data) from repeat samples in inter-plates and intro-plates were individually analyzed through pairwise comparisons to assess the similarity among repeat samples. The matrix of scatterplots indicates the correlation (scatterplots for pairwise samples, in the bottom part of the matrix), sample name (on the diagonal line of the matrix), and Pearson correlation coefficient (R value, in the upper part of the matrix) of each comparison, the higher the R-value is the more correlated for each two pairwise samples.

Figure S2. Effect of Ponatinib on the growth of breast cancer *in vitro* and *in vivo*. (A) Cytotoxicity of Ponatinib determined by MTS-based viability assay. MDA-MB-231 and LM2 cells were treated with 8 point does ranges of Ponatinib for 72 h. The x-axis displays log2 concentration of Ponatinib. Data are mean ± S.D. of three independent experiments. (B) LM2 cells were implanted into the mammary fat pads of nude mice, and the primary tumor growth of vehicle- or Ponatinib-treated mice was measured. n = 5 mice per group. *P* value is based on one-tailed Mann-Whitney U test, and n.s. means not significant. Error bars, SEM.

Figure S3. **Knocking down *ABL* affects the expression of only half of Ponatinib-regulated BCLM-associated genes in LM2 cells.** (A) RT-qPCR analysis of the expression of the indicated genes with control or *ABL1/ABL2* knockdown in LM2 cells. (B) RT-qPCR analysis of the efficiency of *ABL1/ABL2* knockdown. Data are the mean ± S.D. of three independent experiments. (C) The expression of the indicated genes with control, *ABL1/ABL2* or both- knockdown in 1833 cells were quantified (GSE69125).

Figure S4. The expression of 6 BCLM-associated genes, *JUN*, *ABL1* and *ABL2* genes by RNA-seq analysis. (A and B) The UCSC genome browser was used to track and quantify the mRNA levels of these indicated genes from RNA-seq datasets in Ponatinib-treated (A) or *ABL1/ABL2* double-knockdown LM2 cells (B).

Figure S5. GO analysis of RNA-seq data from Ponatinib-treated LM2 cells. All the indicated pathways refer to the BioCarta pathway and canonical pathway database.

Figure S6. Validation of *JUN* knockdown or overexpression efficiency. Western blot was

performed to verify *JUN* knockdown (A) or overexpression efficiency (B and C).

Figure S7. Cell migration was associated with *JUN* expression in breast cancer cells. (A) Suppression of cell migration by knocking down *JUN* in LM2 cells. Representative images of Transwell membranes stained with crystal violet. Data are mean ± S.D. of three independent experiments. Scale bars, 100 μm. (B) Quantification of migrating cells in the Transwell assay in (A).

Figure S8. Cell migration of breast cancer cells treated with Ponatinib is partially rescued by overexpression of c-Jun. (A) The wound width in MDA-MB-231 cells is quantified among the three groups at all time points after overexpression of *c-Jun* and Ponatinib treatment. ***$P < 0.001$ by paired Student's t-test. (B) Cell migration was analyzed by a Transwell assay. LM2 cells were transfected with vehicle or *JUN* for 48 h and treated with DMSO or 500 nmol/L Ponatinib. Cell migration was analyzed by a Transwell assay. Representative images of Transwell membranes stained with crystal violet. Data are mean ± S.D. of three independent experiments. Scale bars, 200 μm. (C) Quantification of migrating cells in the Transwell assay in (B) **(**upper panel**)**. **$P < 0.01$ by two-sided Student's t-test. Western blot was performed to examine the efficiency of *c-Jun* overexpression (lower panel).
