# Supplementary material for: Chemical genomics reveals inhibition of breast cancer lung metastasis by Ponatinib via c-Jun
Source: Protein Cell. 2018 Apr 17;10(3):161–77. doi: 10.1007/s13238-018-0533-8 (PMC6338618; doi:10.1007/s13238-018-0533-8)
Supplement: Supplementary file 2 — Supplementary material 1 (PPTX 1,860 kb) [file 13238_2018_533_MOESM2_ESM.pptx]

## Slide 1
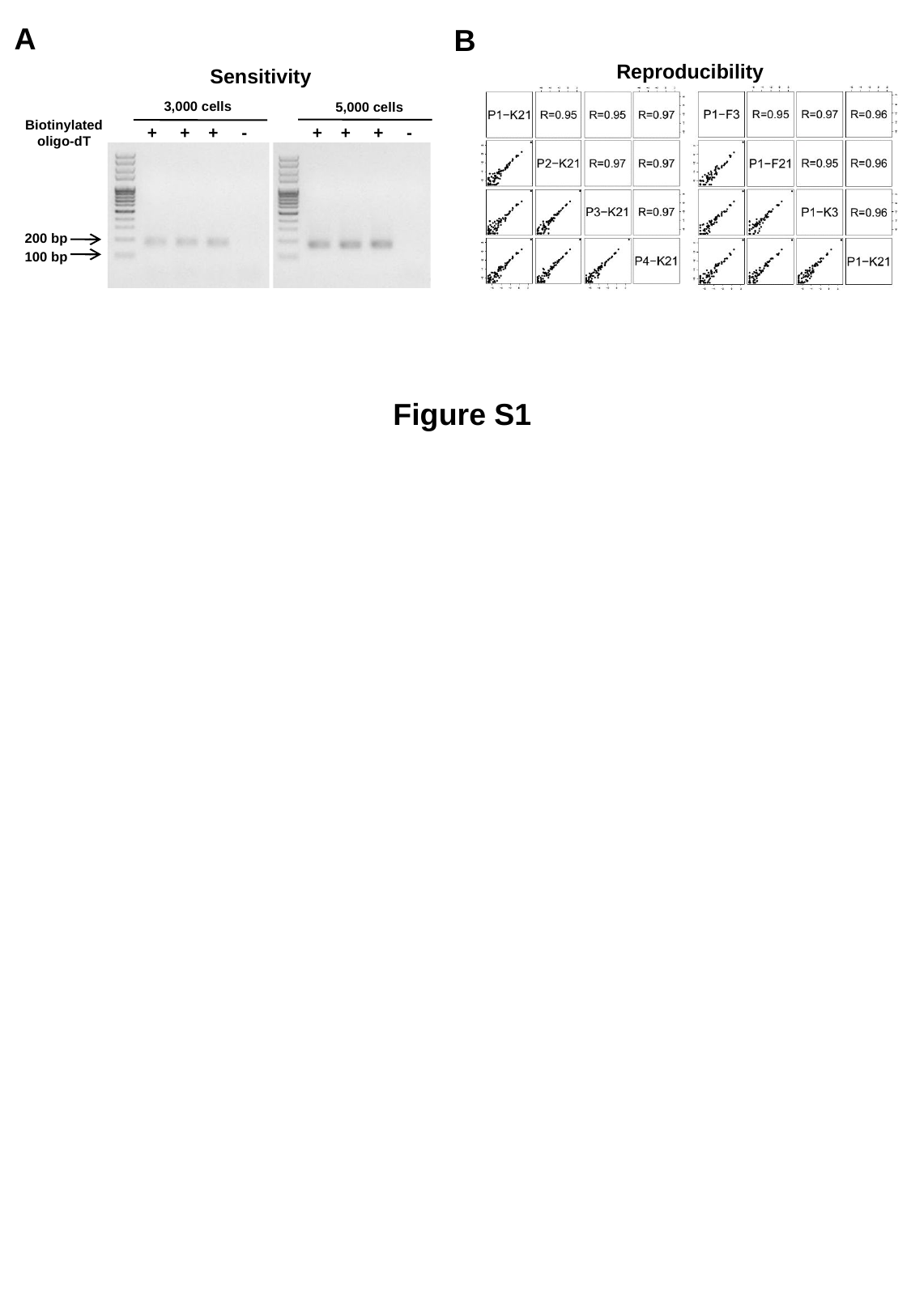

A
B
Reproducibility
Sensitivity
3,000 cells
5,000 cells
Biotinylated oligo-dT
 + + + -
200 bp
100 bp
 + + + -
Figure S1

## Slide 2
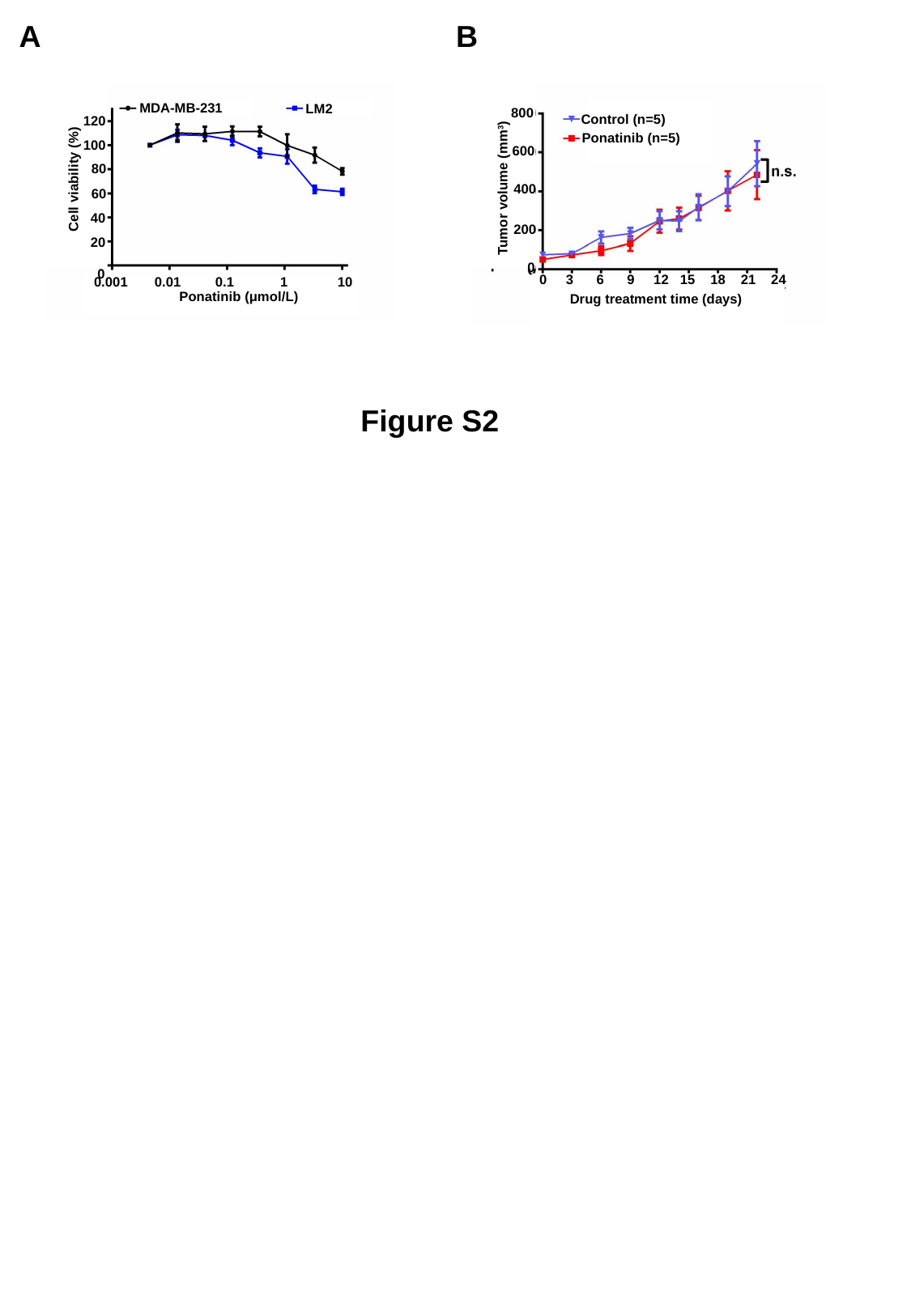

A
B
MDA-MB-231
LM2
800
Control (n=5)
120
Ponatinib (n=5)
100
600
80
Cell viability (%)
Tumor volume (mm3)
400
60
40
200
20
0
0
0 3 6 9 12 15 18 21 24
0.001 0.01 0.1 1 10
Ponatinib (μmol/L)
Drug treatment time (days)
Figure S2
600

## Slide 3
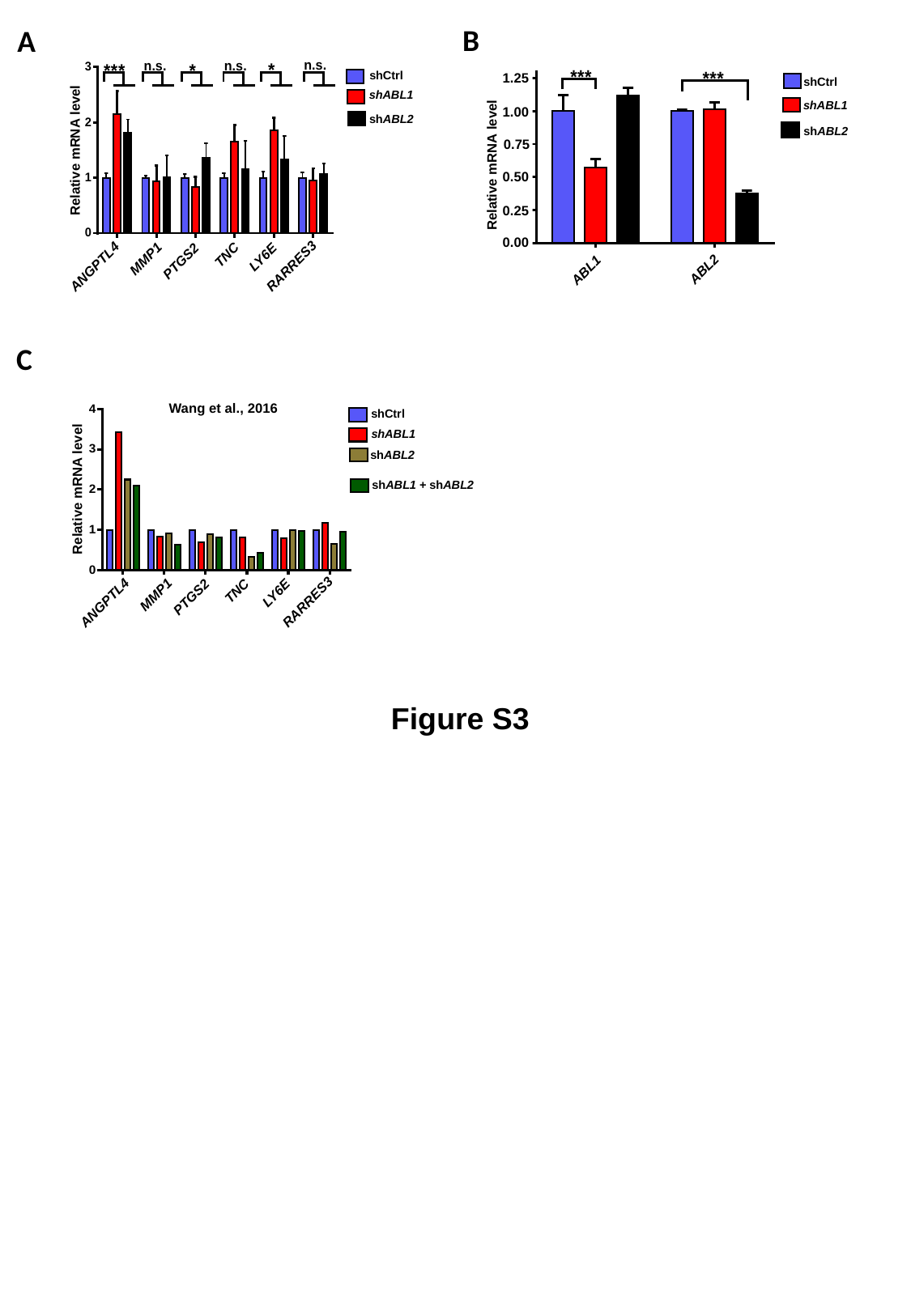

B
A
 shCtrl
1.25
 shCtrl
 shABL1
 shABL1
1.00
 shABL2
 shABL2
Relative mRNA level
0.75
Relative mRNA level
0.50
0.25
TNC
LY6E
0.00
MMP1
PTGS2
ABL2
ABL1
ANGPTL4
RARRES3
C
Wang et al., 2016
 shCtrl
 shABL1
 shABL2
Relative mRNA level
 shABL1 + shABL2
TNC
LY6E
MMP1
PTGS2
ANGPTL4
RARRES3
Figure S3

## Slide 4
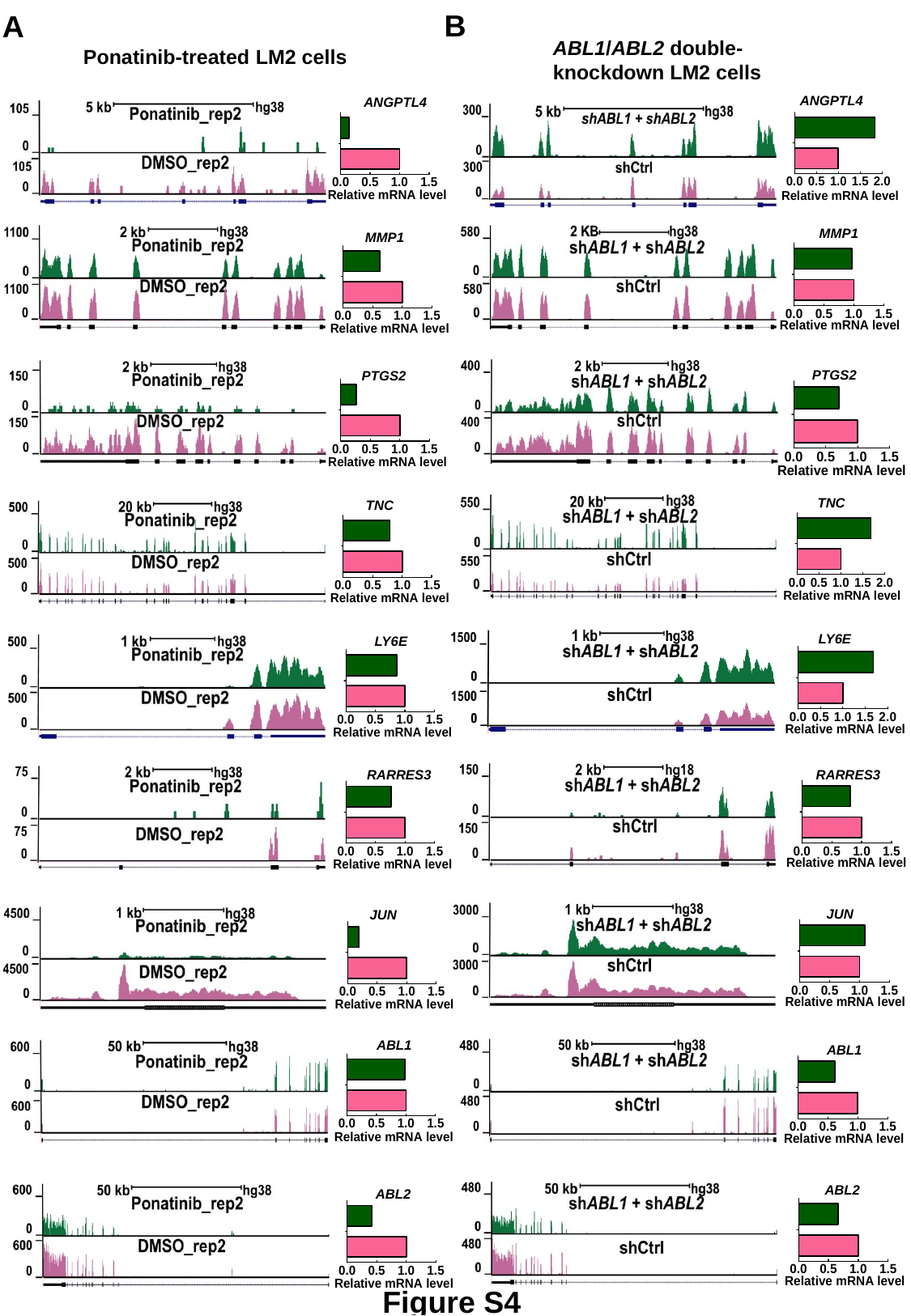

B
A
ABL1/ABL2 double-
knockdown LM2 cells
Ponatinib-treated LM2 cells
ANGPTL4
MMP1
PTGS2
TNC
LY6E
RARRES3
JUN
ABL1
ABL2
ANGPTL4
MMP1
PTGS2
TNC
LY6E
RARRES3
JUN
ABL1
ABL2
 Figure S4

## Slide 5
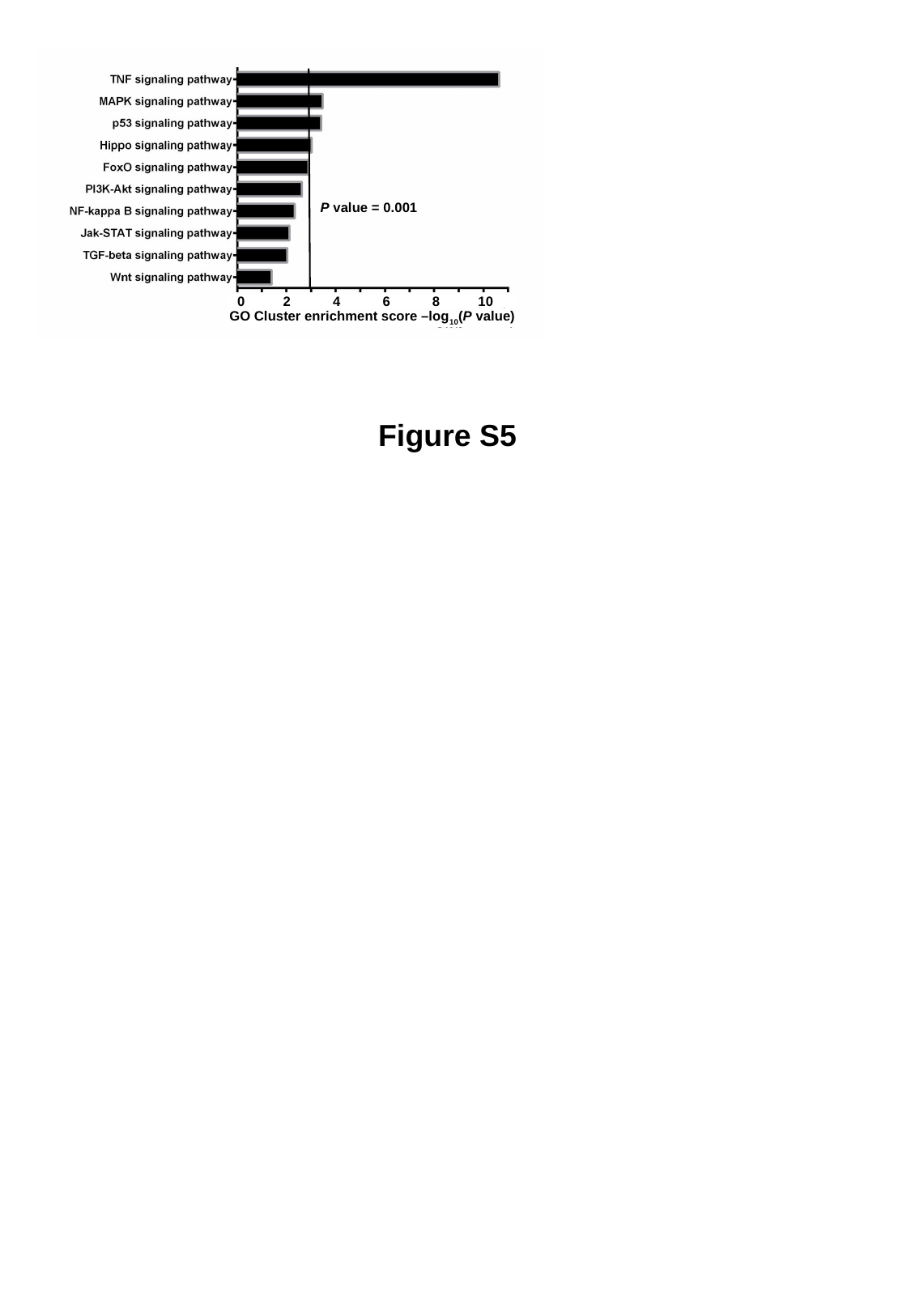

P value = 0.001
0 2 4 6 8 10
GO Cluster enrichment score –log10(P value)
Figure S5

## Slide 6
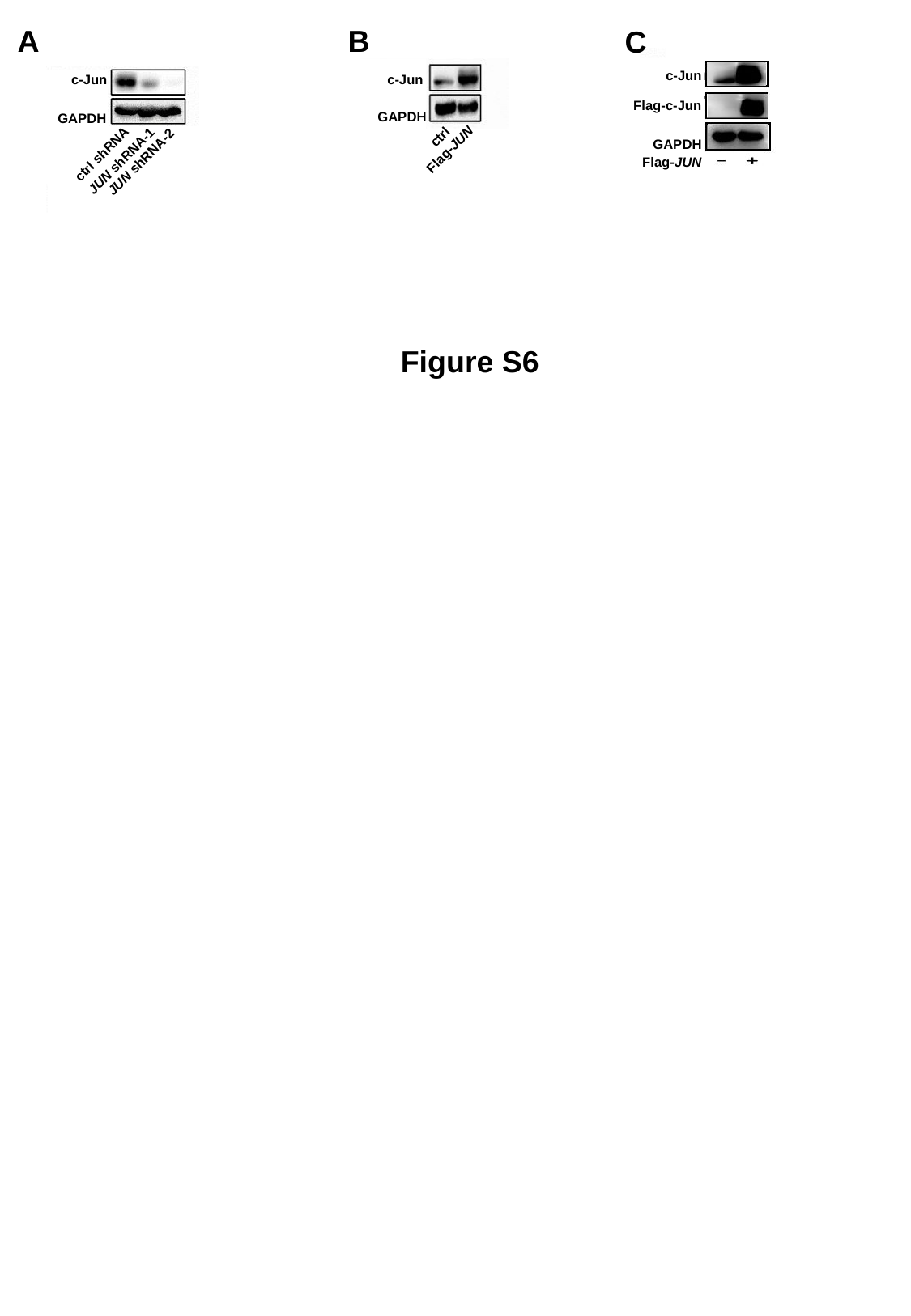

A
B
C
 c-Jun
 c-Jun
 c-Jun
 GAPDH
 GAPDH
 Flag-c-Jun
 GAPDH
 ctrl
 ctrl shRNA
 Flag-JUN
 Flag-JUN
 JUN shRNA-2
 JUN shRNA-1
Figure S6

## Slide 7
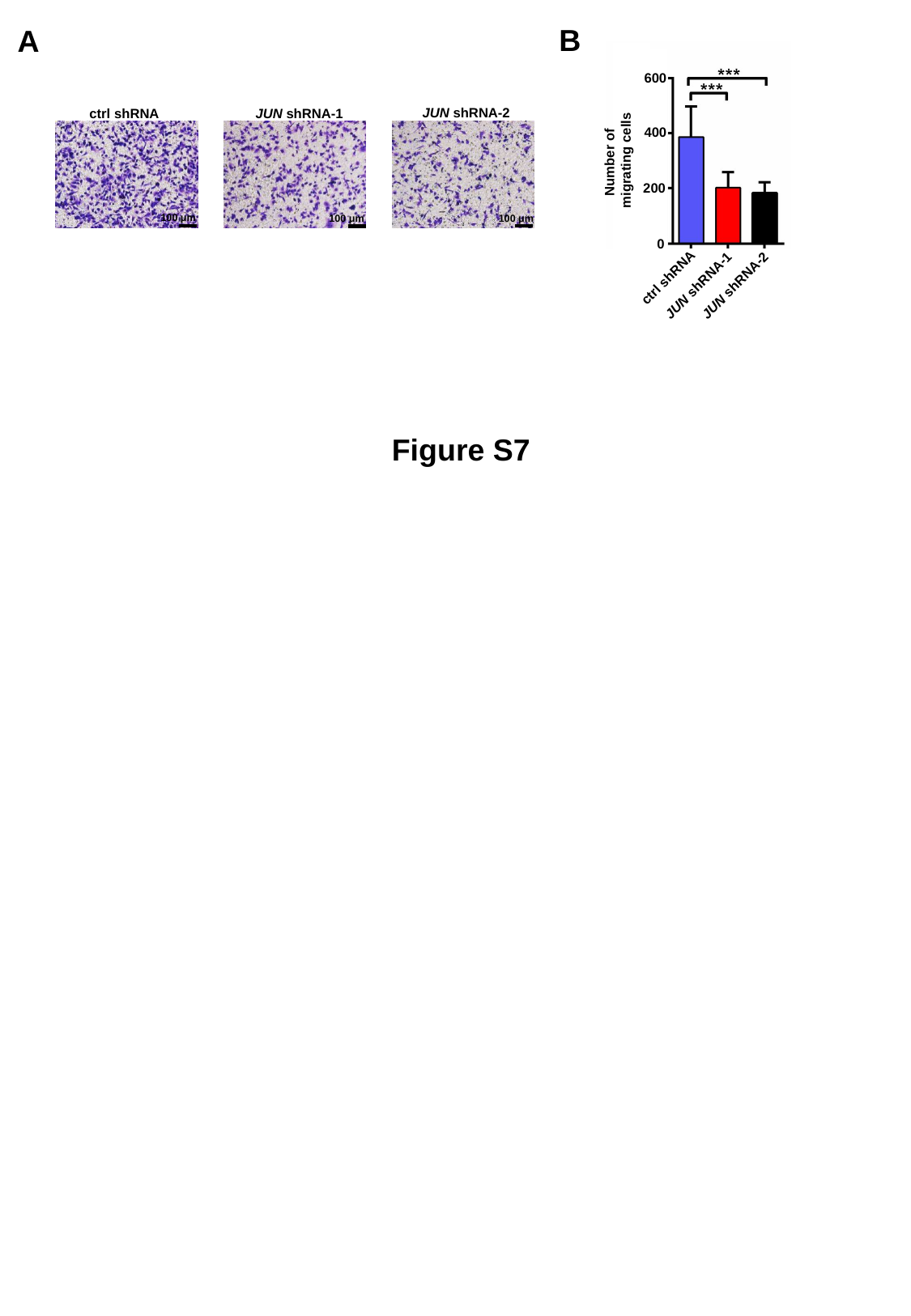

B
A
600
 JUN shRNA-2
 JUN shRNA-1
 ctrl shRNA
100 μm
400
Number of
 migrating cells
200
100 μm
100 μm
 0
 ctrl shRNA
 JUN shRNA-1
 JUN shRNA-2
Figure S7

## Slide 8
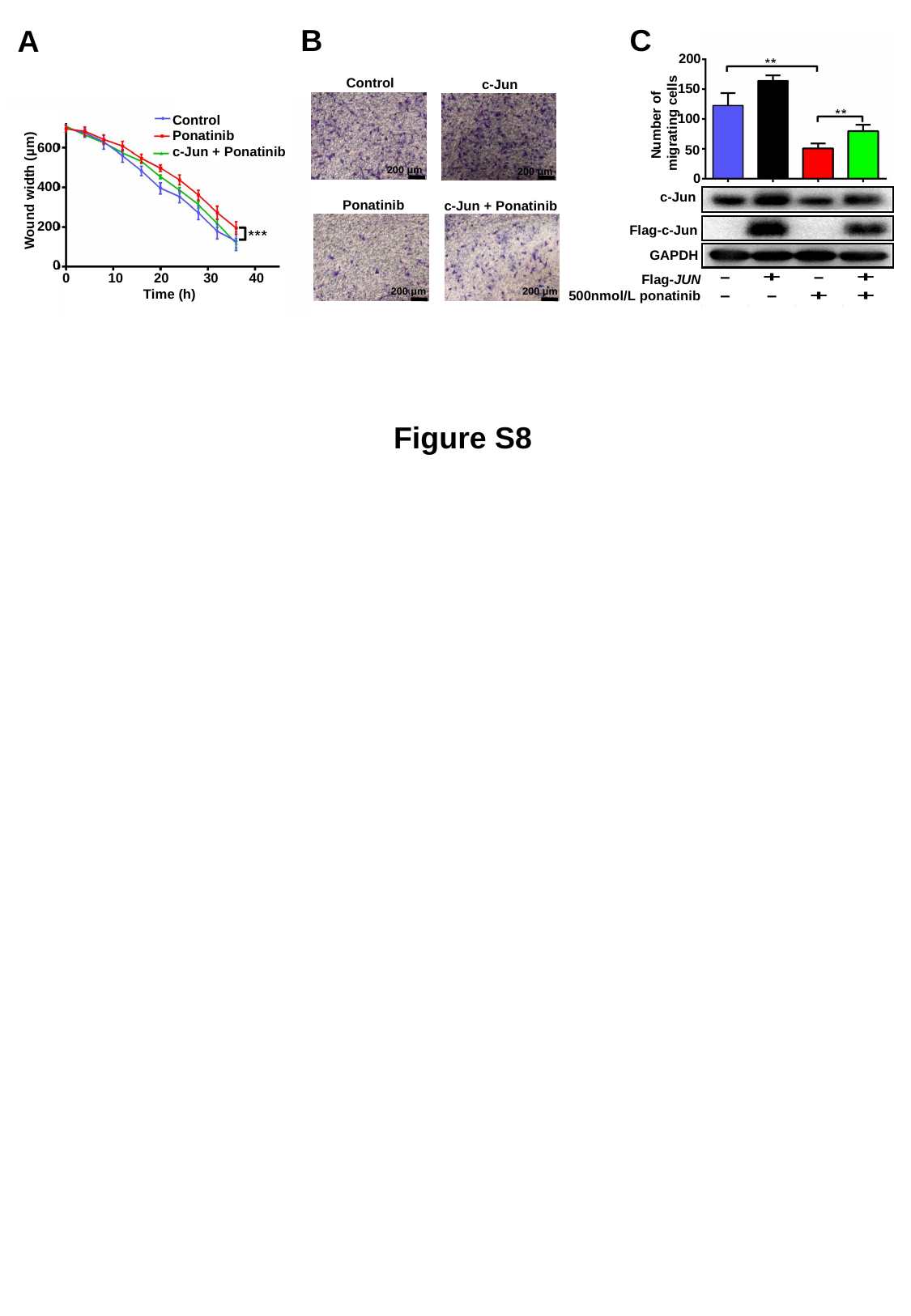

B
C
A
200
Control
c-Jun
Ponatinib
c-Jun + Ponatinib
150
Number of
 migrating cells
100
Control
Ponatinib
c-Jun + Ponatinib
600
50
Wound width (µm)
200 μm
200 μm
0
400
 c-Jun
 Flag-c-Jun
200
 GAPDH
 Flag-JUN
 500nmol/L ponatinib
0
0 10 20 30 40
 Time (h)
200 μm
200 μm
Figure S8
